# Association between sleep duration and mortality risk among adults with type 2 diabetes: a prospective cohort study

**DOI:** 10.1007/s00125-020-05214-4

**Published:** 2020-07-16

**Authors:** Yafeng Wang, Wentao Huang, Adrienne O’Neil, Yutao Lan, Dagfinn Aune, Wei Wang, Chuanhua Yu, Xiong Chen

**Affiliations:** 1 Department of Epidemiology and Biostatistics, School of Health Sciences, Wuhan University, 185 Donghu Road, Wuchang District, Wuhan, 430071 Hubei China; 2 School of Nursing, Guangdong Pharmaceutical University, Guangzhou, China; 3 The Centre for Innovation in Mental and Physical Health and Clinical Treatment, Deakin University, Geelong, VIC Australia; 4 Melbourne School of Population and Global Health, University of Melbourne, Carlton, VIC Australia; 5 Department of Epidemiology and Biostatistics, School of Public Health, Imperial College London, London, UK; 6 Department of Nutrition, Bjørknes University College, Oslo, Norway; 7 Department of Endocrinology, Morbid Obesity and Preventive Medicine, Oslo University Hospital, Oslo, Norway; 8 School of Mathematical Sciences, Shanghai Jiao Tong University, Shanghai, China; 9 Department of Endocrinology, The First Affiliated Hospital of Wenzhou Medical University, Wenzhou, 325000 Zhejiang China

**Keywords:** Cohort study, Diabetes mellitus, Mortality, Sleep duration

## Abstract

**Aims/hypothesis:**

This study aimed to investigate whether the effects of sleep duration interacted with the presence of diabetes. We specifically sought to examine the relationship between sleep duration and all-cause and cause-specific mortality in people with type 2 diabetes across sex, age at diagnosis, duration of diabetes and treatment type.

**Methods:**

The sample consisted of 273,029 adults, including 248,817 without diabetes and 24,212 with type 2 diabetes, who participated in the National Health Interview Survey from 2004 to 2013 and whose data were linked to a mortality database up to 31 December 2015. Sleep duration was measured using self-report, whereby participants were asked ‘on average how long do you sleep each day (≤5, 6, 7, 8, 9 or ≥10 h/day)?’ The relationship between sleep duration and mortality risk was investigated using Cox proportional hazards regression model, with adjustments for demographics, BMI, lifestyle behaviours and clinical variables.

**Results:**

Absolute mortality rate was higher in adults with diabetes and extremes of sleep duration (≤5 h/day, 215.0 per 10,000 person-years; ≥10 h/day, 363.5 per 10,000 person-years). There was a non-significant interaction between sleep duration and the presence of diabetes (*p* for interaction = 0.08). A J-shaped relationship existed between sleep duration and all-cause mortality risk in people with type 2 diabetes. Compared with the reference group (7 h/day), both shorter and longer sleep durations were associated with increased risk of all-cause mortality (≤5 h/day, HR 1.24 [95% CI 1.09, 1.40]; 6 h/day, HR 1.13 [1.01, 1.28]; 8 h/day, HR 1.17 [1.06, 1.30]; ≥10 h/day, HR 1.83 [1.61, 2.08]). Similar associations were also observed for mortality risk from CVD, cancer, kidney disease, Alzheimer’s disease and chronic lower respiratory diseases. Longer sleep duration in those with a younger age at diabetes onset was associated with greater risks of all-cause and CVD mortality. Shorter sleep duration in individuals treated with both insulin and oral glucose-lowering medication was also associated with higher risks of all-cause and CVD mortality.

**Conclusions/interpretation:**

The associations between sleep duration and mortality risk may be different between diabetic and non-diabetic individuals. In people with type 2 diabetes, sleeping less or more than 7 h/day was associated with increased risk of all-cause and condition-specific mortality. The association was more prominent in those with a younger age at diabetes onset and receiving treatment with both oral glucose-lowering medication and insulin. This population may benefit from targeted sleep-related interventions to reduce the risks of adverse health outcomes.

**Figure:**
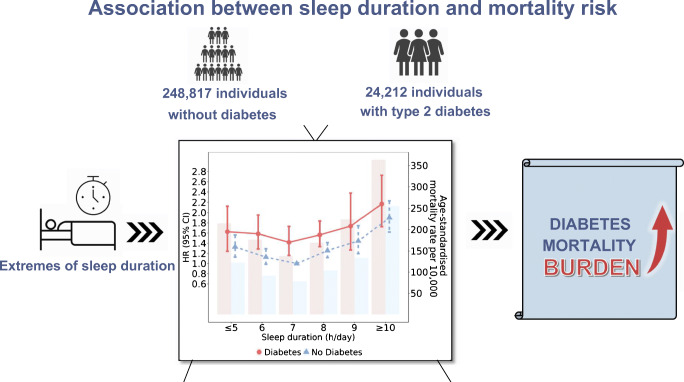
Graphical abstract

**Electronic supplementary material:**

The online version of this article (10.1007/s00125-020-05214-4) contains peer-reviewed but unedited supplementary material, which is available to authorised users.

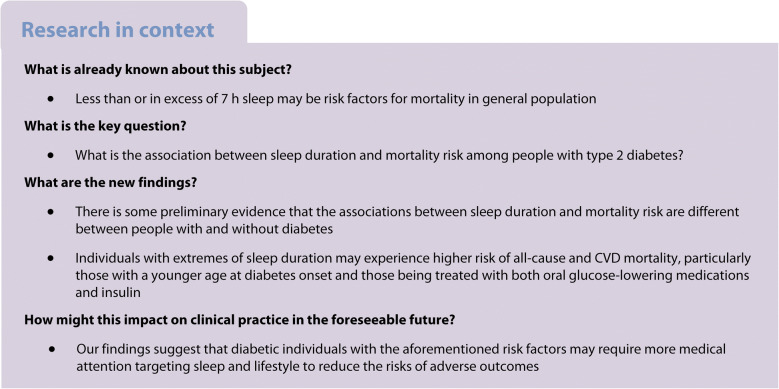



## Introduction

Diabetes is a worldwide public health crisis. Globally, the prevalence of diabetes was 8.8% in 2017, affecting an estimated 424.9 million people [1]. This number is predicted to increase to 628.6 million by 2045 [1]. Diabetes was the seventh highest cause of premature death in 2016 [1] and is associated with an increased risk of all-cause mortality as well as condition-specific morbidity and mortality including CVD, cancer and kidney disease [2–7]. CVD is among the foremost diabetes-related causes of death [1, 8].

A recent study showed that death rates have obvious differences between people with diabetes and those without [9]. In addition, an earlier study found that there are weaker relative educational disparities in mortality among adults with vs those without diabetes [10]. The benefit from physical activity in individuals with CVD was shown to be greater than that in healthy individuals without CVD [11]. Mounting epidemiological evidence suggests that sleep duration is related to increased risks of CVD events and higher mortality risk in the general population [12–16]. However, no previous studies have compared the effect of sleep duration on mortality risk among participants with and without diabetes.

In addition, both insulin treatment and metformin therapy are associated with sleep duration and quality in individuals with type 2 diabetes [17, 18]. It is well established that individuals who experience short sleep duration and poor sleep quality have a higher risk of obesity, glucose intolerance, insulin resistance and poor glycaemic control, which would directly and indirectly affect outcomes among those with diabetes [19]. Moreover, studies have suggested that sex difference in the mortality rate associated with diabetes and age at onset of type 2 diabetes is a prognostically important determinant for survival outcomes [20, 21]. The role of sleep in this association remains unclear; although, it is biologically plausible that too little or too much sleep in people with type 2 diabetes would lead to poorer survival outcomes. Therefore, it is necessary to investigate the association of sleep duration with all-cause and disease-specific mortality in people with type 2 diabetes across the above subgroups.

Using data from the National Health Interview Survey (NHIS) in the USA, the aim of this study was to investigate whether the effect of sleep duration interacted with the presence of diabetes. We specifically sought to examine the relationship between sleep duration and all-cause and cause-specific mortality in people with type 2 diabetes across sex, age at diagnosis, duration of diabetes and treatment.

## Methods

### Participants

The NHIS is a nationally representative and multistage stratified survey of non-institutionalised individuals in the USA. In the survey, one adult and one child are selected randomly from each household for a detailed interview on health status and lifestyle behaviours. Details of the survey design and methods are displayed at the website of NHIS from National Center for Health Statistics (NCHS) (www.cdc.gov/nchs/nhis/about_nhis.htm; accessed 7 January 2018). NHIS was approved by the NCHS ethics review board. We could not influence the design of the prior studies upon which this work is based and cannot comment on individual ethics approval or consent. The data we used are publicly available and considered as exempt under the ethical board review of the corresponding author’s institution.

A total of 289,187 adults who participated in the NHIS from 2004 to 2013 were eligible to be linked to a mortality database over the follow-up period (up to 31 December 2015). After excluding missing data about sleep duration (*n* = 14,382), diabetes (*n* = 148), age at diagnosis of diabetes (*n* = 497) or use of oral glucose-lowering medication (*n* = 18) or insulin (*n* = 4), 274,138 remained. Among those with diabetes, we excluded individuals with possible type 1 diabetes (*n* = 1109), defined by use of insulin and age at diabetes onset <30 years (validated as accurate in 97% of cases [9]); the remaining 24,212 individuals were defined as having type 2 diabetes. The total study population of 273,029 adults also included 248,817 without diabetes. NHIS were reviewed and approved by the NCHS Research Ethics Review Board, and Institutional Review Board approval was not required because this study was based on secondary analyses of publicly available, de-identified data.

### Study outcomes: mortality

Mortality outcomes were determined by the National Death Index (NDI) records. Accuracy of the all-cause and cause-specific death information and consistency of the matching algorithm in the NDI records were verified [22]. The underlying causes of death were collected and classified according to the International Classification of Diseases (ICD-10) guidelines (http://apps.who.int/classifications/icd10/browse/2016/en). In addition to all-cause mortality, we were interested in the following cause-specific mortality outcomes as they are the eight leading causes of death in the general population: CVD, including heart disease and stroke; cancer; chronic lower respiratory disease (CLRD); Alzheimer’s disease; diabetes mellitus; influenza and pneumonia; and kidney disease. Electronic supplementary material (ESM) Table 1 shows specific codes for causes of death.

### Study exposure: sleep duration

Sleep duration data were obtained from the following self-reported interview question: ‘On average, how many hours of sleep do you get in a 24 h period?’. The smallest unit of increments was 1 h, and sleep duration was subsequently categorised into six groups (≤5, 6, 7, 8, 9 and ≥10 h/day). Extreme sleep duration was defined as sleep duration ≤5 h/day or ≥10 h/day.

### Covariates

The following covariates were included as adjustment variables: age; sex; race (Hispanic, non-Hispanic White, non-Hispanic black, and others); education level (less than high school degree, high school degree, and more than high school degree); household income; lifestyle variables of BMI (normal weight or underweight [<25 kg/m^2^], pre-obese [25 kg/m^2^ to ≤30 kg/m^2^], obese [>30 kg/m^2^]), physical activity (meeting or not the 2008 Physical Activity Guidelines for Americans that recommend at least 75 min of vigorous physical activity or 150 min of moderate physical activity in 1 week), smoking status (non-smoker, former smoker, current smoker), alcohol drinking status (lifetime abstainer, former drinker, current drinker); clinical variables with self-reported diagnoses of hypertension (e.g. BP), CHD, stroke and cancer; and calendar year. We also collected and categorised the duration of diabetes into four groups of 0–5, 6 to ≤10, 11 to ≤20 and >20 years. Age at diagnosis of diabetes was classified into two groups (≤45 or >45 years of age [young and old, respectively]) [23].

Medication status was stratified into four categories: insulin only, oral glucose-lowering medication only, both and no medication.

### Statistical analysis

Descriptive statistics were used to report the distribution of participants’ baseline characteristics by sleep duration. Continuous variables were displayed as mean ± SE, and categorical variables were displayed as percentage (%). Continuous data were compared with analysis of *t* test and variance, while Pearson’s *χ*^2^ tests were used to compare differences in baseline characteristics (including the exposure and covariates). Mortality rates were age-standardised to the overall NHIS using the age groups of 18–44 years, 45–64 years and 65 years or older. The graphical assessment of log–log plots was used to assess the proportional hazards assumption and the assumption was met in each of the models [24]. We used a multivariate Cox proportional hazards regression model to estimate HRs with corresponding 95% CIs to test the association between sleep duration categories and all-cause and cause-specific mortality. The reference group was 7 h/day. In multivariate models, we adjusted for age (as a timescale), sex, race, education level, household income, duration of diabetes (years), BMI, physical activity, smoking status, alcohol drinking status, hypertension, CHD, stroke, cancer and calendar year. Moreover, we also conducted subgroup analyses for all-cause and CVD-related mortality stratified by sex, duration of diabetes and age at diagnosis. Adjusted Wald test accounting for complex multistage sampling design was used to test for interaction term. To examine the robustness of the results, we performed sensitivity analyses excluding individuals with a history of CHD and stroke or cancer. Sampling weights were used to account for the unequal probabilities of selection. SEs were calculated by Taylor series linearisation. A *p* value <0.05 was considered statistically significant (two-tailed). All data analyses were performed using STATA version 12.0 (Stata Corp, College Station, TX, USA).

## Results

### Participant characteristics

Among the 248,817 adults without diabetes, there were 17,060 deaths during the mean 6.70 years of follow-up (1.62 million person-years). A total of 4593 deaths (2331 for women, 2262 for men) were recorded during a mean follow-up of 5.96 years (0.14 million person-years) among the 24,212 participants with diabetes. Baseline characteristics of participants with or without diabetes by sleep duration are presented in Table 1 and ESM Table 2, respectively.

**Table 1:** Baseline characteristics among adults with type 2 diabetes according to sleep duration

Characteristic	Sleep duration (h/day)					
	≤5	6	7	8	9	≥10
Sample size, *n* (%)	2957 (12.2)	5054 (20.9)	5379 (22.2)	7497 (31.0)	1341 (5.5)	1984 (8.2)
Mean age, years	59.1	59.8	60.4	62.9	65.9	66.3
Sex, *n* (%)						
Male	1179 (43.9)	2238 (49.0)	2538 (51.9)	3535 (51.4)	601 (48.3)	888 (48.8)
Female	1778 (56.1)	2816 (51.1)	2841 (48.2)	3962 (48.6)	740 (51.7)	1096 (51.2)
Race, *n* (%)						
Hispanic	572 (14.7)	895 (14.0)	987 (14.2)	1416 (14.6)	182 (10.5)	313 (12.5)
Non-Hispanic white	1464 (60.2)	2684 (62.9)	3049 (65.8)	4201 (65.4)	859 (73.4)	1143 (68.2)
Non-Hispanic black	756 (20.1)	1143 (16.7)	1014 (13.9)	1509 (14.7)	238 (12.0)	440 (15.3)
Non-Hispanic other	165 (5.0)	332 (6.5)	329 (6.1)	371 (5.3)	62 (4.1)	88 (4.0)
Education level, *n* (%)						
Less than high school degree	894 (26.7)	1248 (21.7)	1204 (18.5)	2104 (24.6)	377 (24.8)	752 (35.4)
High school degree	865 (30.6)	1491 (30.8)	1580 (30.8)	2310 (32.0)	429 (32.9)	582 (31.8)
More than high school degree	1180 (42.1)	2291 (47.2)	2577 (50.4)	3037 (42.8)	529 (41.9)	629 (32.0)
Household income, *n* (%)						
Low	873 (23.8)	1002 (14.3)	857 (11.6)	1414 (13.9)	250 (13.5)	497 (18.9)
Middle	1604 (55.6)	2778 (55.7)	2843 (50.5)	4320 (57.4)	799 (59.8)	1211 (62.7)
High	480 (20.6)	1274 (30.1)	1679 (37.9)	1763 (28.8)	292 (26.7)	276 (18.4)
BMI, *n* (%)						
Normal weight/underweight (<25 kg/m^2^)	412 (12.8)	721 (13.0)	779 (13.6)	1269 (16.3)	252 (17.7)	376 (18.9)
Pre-obese (25–30 kg/m^2^)	786 (26.1)	1463 (27.8)	1721 (32.0)	2421 (32.7)	431 (32.3)	561 (27.6)
Obese (>30 kg/m^2^)	1668 (58.4)	2741 (56.2)	2737 (51.5)	3561 (47.8)	630 (47.6)	994 (51.1)
Meeting physical activity requirements, *n* (%)	686 (23.6)	1409 (29.6)	1738 (35.0)	2158 (29.9)	349 (27.7)	287 (15.0)
Smoking status, *n* (%)						
Never	1425 (47.0)	2559 (48.7)	2850 (51.7)	3868 (50.9)	627 (45.4)	908 (44.8)
Previous	852 (28.9)	1635 (34.1)	1753 (33.2)	2590 (35.3)	510 (40.2)	739 (39.3)
Current	677 (24.0)	850 (17.1)	769 (14.7)	1028 (13.7)	299 (14.1)	334 (15.8)
Alcohol drinking status, *n* (%)						
Never	865 (27.0)	1394 (25.3)	1484 (24.7)	2207 (27.6)	388 (26.0)	617 (29.3)
Previous	905 (30.3)	1454 (28.5)	1431 (25.1)	2247 (29.6)	452 (32.6)	776 (38.1)
Current	1162 (41.6)	2171 (45.7)	2415 (49.3)	2986 (41.9)	498 (41.3)	581 (32.2)
Hypertension, *n* (%)	2238 (72.9)	3720 (71.7)	3746 (67.5)	5353 (69.6)	1014 (74.4)	1549 (76.1)
CHD, *n* (%)	524 (18.2)	821 (15.0)	730 (13.4)	1279 (17.3)	273 (20.9)	464 (24.2)
Stroke, *n* (%)	312 (10.0)	418 (7.5)	358 (5.8)	706 (9.2)	183 (13.7)	362 (18.9)
Cancer, *n* (%)	454 (15.5)	713 (14.3)	784 (14.0)	1109 (14.6)	248 (19.2)	381 (20.3)
Diabetes duration, *n* (%)						
0–5 years	998 (37.3)	1686 (35.1)	1898 (37.7)	2407 (33.5)	388 (29.6)	488 (25.9)
6 – ≤10 years	671 (22.5)	1165 (23.3)	1264 (24.2)	1703 (23.0)	286 (22.5)	407 (21.1)
11 – ≤20 years	798 (26.0)	1328 (25.4)	1380 (24.8)	1965 (25.9)	363 (26.1)	561 (28.3)
>20 years	490 (14.2)	872 (16.3)	837 (13.3)	1422 (17.6)	304 (21.8)	528 (24.8)
Age at diabetes diagnosis, *n* (%)						
0–45 years	1217 (42.3)	2016 (43.0)	1937 (37.4)	2518 (35.2)	414 (31.0)	658 (34.9)
>45 years	1740 (57.7)	3038 (57.0)	3442 (62.6)	4979 (64.8)	927 (69.1)	1326 (65.2)

The NHIS used a multistage area probability sampling design. Analyses of per cent values presented in this table were conducted using the final weights, which represent a product of weights for corresponding units computed in each of the sampling stages. See the statistical analysis section of the Methods for details

### Impact of sleep duration on mortality

**Fig. 1:**
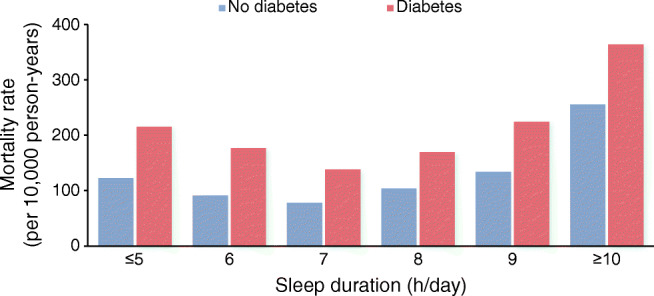
Age-standardised mortality rates according to sleep duration among adults with and without type 2 diabetes

Figure 1 shows age-standardised mortality rate per 10,000 person-years stratified by the presence of diabetes and sleep duration. Mortality rate was highest with extreme sleep duration (≤5 h/day and ≥10 h/day) and lowest with sleep duration of 7 h/day, regardless of the presence of type 2 diabetes (Table 2).

**Table 2:** Sleep duration and the risk of mortality stratified by the presence of type 2 diabetes

Sleep duration, h/day	Participants, *n*	Deaths, *n*	Age-standardised mortality rate (per 10,000 person-years)	Model 1^a^		Model 2^b^	
				HR (95% CI)	*p* value	HR (95% CI)	*p* value
No diabetes							
≤5	20,676	1563	122.2	1.74 (1.48, 1.79)	<0.001	1.33 (1.13, 1.56)	0.001
6	52,316	2788	91.2	1.27 (1.11, 1.46)	<0.001	1.13 (0.99, 1.29)	0.08
7	75,182	3659	78.3	1 (reference)	–	1 (reference)	–
8	79,669	5793	103.6	1.36 (1.21, 1.53)	<0.001	1.26 (1.12, 1.41)	<0.001
9	11,313	1281	133.8	1.65 (1.36, 1.99)	<0.001	1.45 (1.20, 1.75)	<0.001
≥10	9661	1976	255.5	2.72 (2.32, 3.20)	<0.001	1.90 (1.62, 2.23)	<0.001
Type 2 diabetes							
≤5	2957	521	215.0	2.78 (2.14, 3.61)	<0.001	1.63 (1.24, 2.13)	<0.001
6	5054	806	176.8	2.37 (1.93, 2.93)	<0.001	1.59 (1.29, 1.95)	<0.001
7	5379	779	138.0	1.84 (1.51, 2.34)	<0.001	1.42 (1.16, 1.73)	0.001
8	7497	1468	169.4	2.20 (1.87, 2.58)	<0.001	1.56 (1.33, 1.84)	<0.001
9	1341	348	224.2	2.58 (1.90, 3.51)	<0.001	1.74 (1.27, 2.38)	0.001
≥10	1984	671	363.5	3.67 (2.93, 4.60)	<0.001	2.17 (1.72, 2.73)	<0.001

^a^Model 1: adjusted for age and sex

^b^Model 2: Model 1, additionally adjusted for race/ethnicity, education, household income, BMI, smoking, alcohol intake, physical activity, CHD, stroke and cancer

**Fig. 2:**
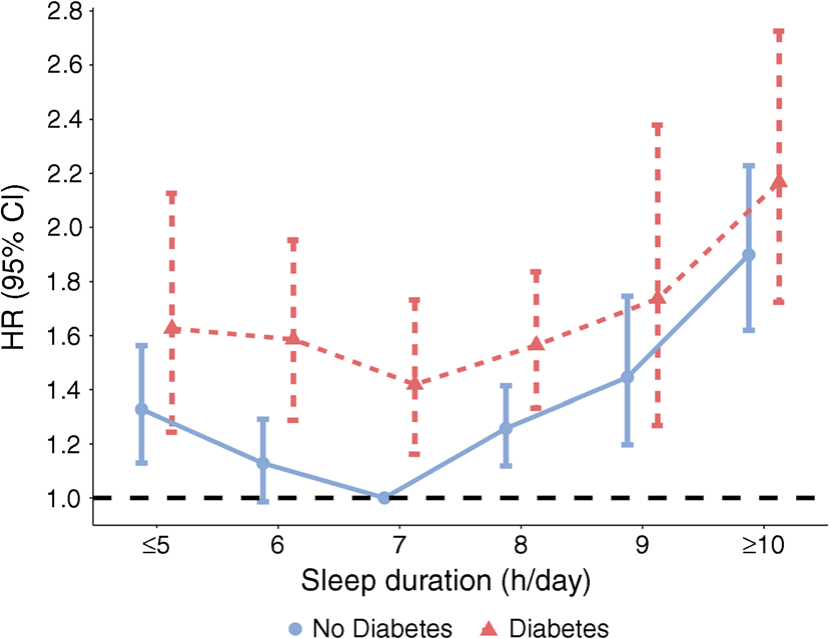
Adjusted mortality risk according to sleep duration stratified by type 2 diabetes

Extremes of sleep duration were associated with increased risk in mortality among adults with and without diabetes (Fig. 2). Individuals with type 2 diabetes who reported the shortest and longest sleep duration had higher all-cause mortality risk than the non-diabetic group who slept for 7 h/day (≤5 h/day, HR 1.63 [95% CI 1.24, 2.13]); ≥10 h/day, HR 2.17 [1.72, 2.73]) (Table 2). There was a non-significant interaction between sleep duration and the presence of diabetes (*p* for interaction = 0.08).

**Fig. 3:**
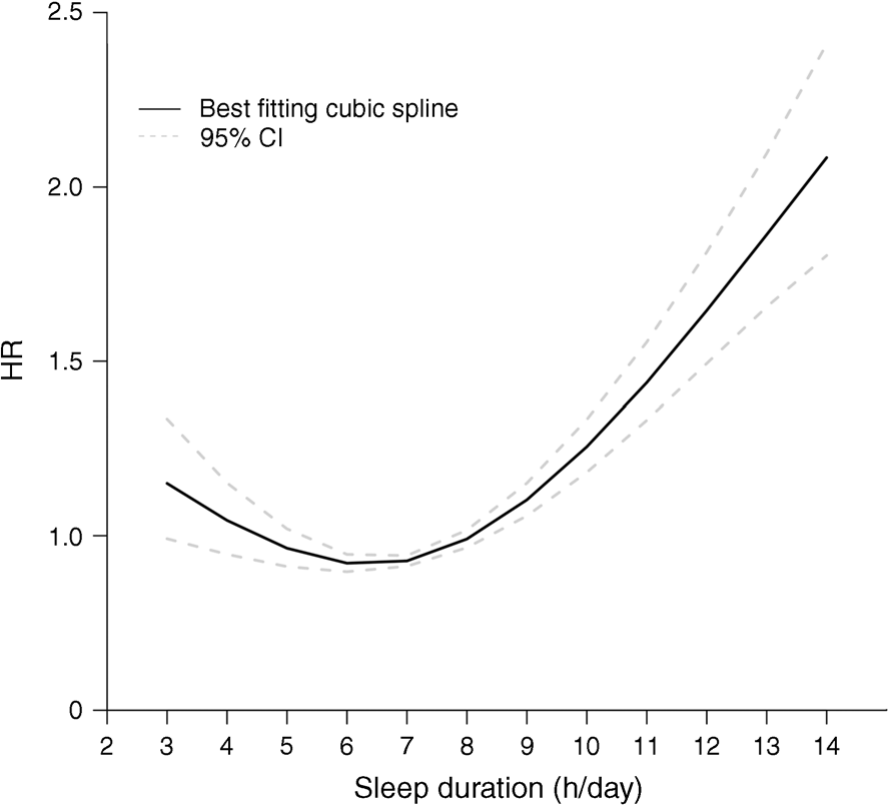
Non-linear dose–response analyses of sleep duration and risk of all-cause mortality among adults with type 2 diabetes

**Fig. 4:**
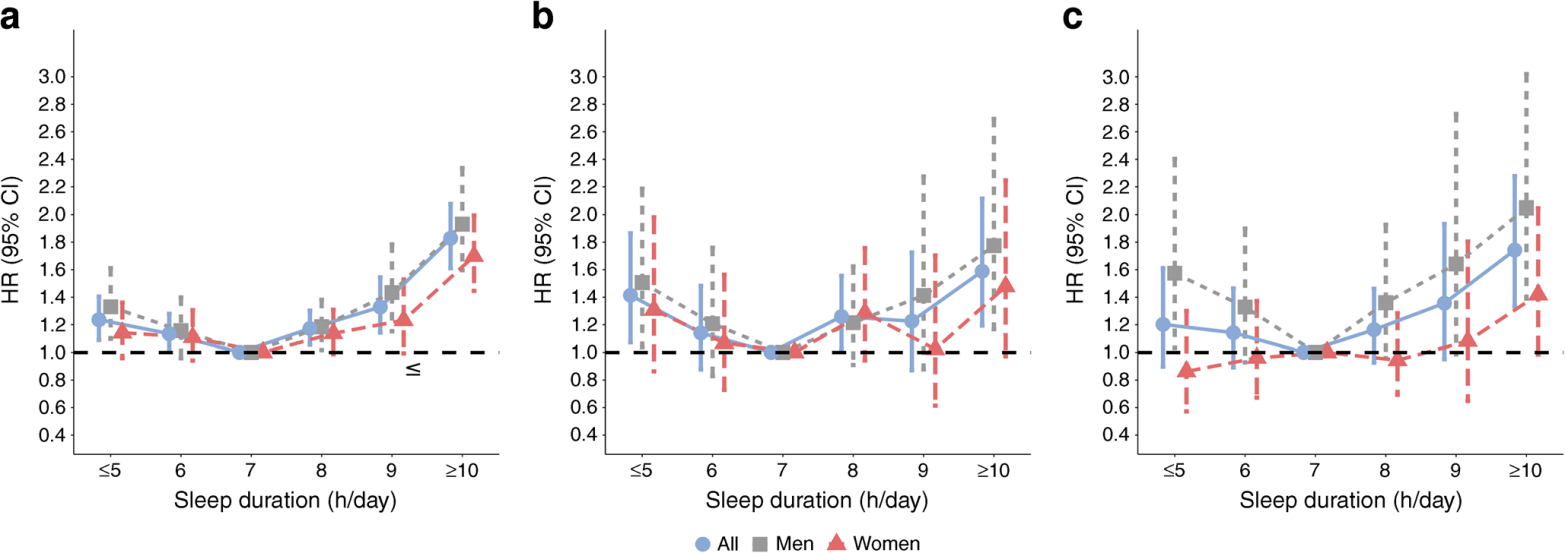
Association of sleep duration with (**a**) all-cause mortality, (**b**) CVD mortality and (**c**) cancer mortality in men and women with type 2 diabetes

Among individuals with type 2 diabetes, compared with the reference group (7 h/day sleep), both shorter and longer sleep duration were associated with increased mortality risk (≤5 h/day, HR 1.24 [1.09, 1.40]; ≥10 h/day, HR 1.83 [1.61, 2.08]) (Table 3 and Fig. 3). A subgroup analysis showed a similar J-shaped curve among women and men, respectively (Table 3 and Fig. 4a).

**Table 3:** Associations of sleep duration with all-cause and cause-specific mortality among individuals with type 2 diabetes

Cause of mortality	Participants, *n*	Deaths, *n*	Mortality risk according to sleep duration, HR (95% CI)						*p* trend	*p* interaction^a^
			≤5 h/day	6 h/day	7 h/day	8 h/day	9 h/day	≥10 h/day		
All-cause mortality										
Total	24,212	4593	1.24 (1.09, 1.40)	1.13 (1.01, 1.28)	1 (reference)	1.17 (1.06, 1.30)	1.33 (0.98, 1.50)	1.83 (1.61, 2.08)	<0.001	
Women	13,233	2331	1.14 (0.96, 1.36)	1.11 (0.94, 1.31)	1 (reference)	1.14 (0.99, 1.31)	1.23 (0.99, 1.53)	1.70 (1.45, 1.99)	<0.001	0.87
Men	10,979	2262	1.33 (1.10, 1.61)	1.21 (0.83, 1.76)	1 (reference)	1.18 (1.02, 1.38)	1.43 (1.15, 1.78)	1.93 (1.60, 2.34)	<0.001	
CVD mortality										
Total	24,212	975	1.20 (0.90, 1.61)	1.14 (0.89, 1.46)	1 (reference)	1.16 (0.93, 1.46)	1.36 (0.95, 1.93)	1.74 (1.33, 2.28)	0.005	
Women	13,233	456	0.86 (0.57, 1.30)	0.96 (0.67, 1.37)	1 (reference)	0.94 (0.69, 1.28)	1.08 (0.65, 1.80)	1.42 (0.99, 2.04)	0.03	0.41
Men	10,979	519	1.57 (1.03, 2.40)	1.33 (0.93, 1.90)	1 (reference)	1.36 (0.96, 1.93)	1.64 (0.99, 2.73)	2.05 (1.39, 3.02)	0.08	
Cancer mortality										
Total	24,212	995	1.41 (1.07, 1.86)	1.14 (0.88, 1.48)	1 (reference)	1.26 (1.02, 1.56)	1.23 (0.87, 1.71)	1.59 (1.19, 2.21)	0.21	
Women	13,233	478	1.31 (0.86, 1.98)	1.07 (0.73, 1.56)	1 (reference)	1.29 (0.94, 1.76)	1.02 (0.61, 1.70)	1.48 (0.97, 2.25)	0.43	0.85
Men	10,979	517	1.51 (1.04, 2.19)	1.21 (0.83, 1.76)	1 (reference)	1.22 (0.91, 1.62)	1.41 (0.88, 2.27)	1.77 (1.17, 2.69)	0.30	
Heart disease mortality										
Total	24,212	779	1.13 (0.81, 1.58)	1.03 (0.77, 1.38)	1 (reference)	1.10 (0.85, 1.43)	1.29 (0.88, 1.90)	1.51 (1.11, 2.07)	0.034	
Women	13,233	340	0.74 (0.45, 1.20)	0.88 (0.57, 1.35)	1 (reference)	0.88 (0.62, 1.26)	1.06 (0.59, 1.91)	1.16 (0.72, 1.86)	0.11	0.49
Men	10,979	439	1.48 (0.94, 2.33)	1.17 (0.78, 1.74)	1 (reference)	1.25 (0.86, 1.83)	1.46 (0.85, 2.49)	1.79 (1.18, 2.73)	0.17	
Stroke mortality										
Total	24,212	196	1.64 (0.83, 3.24)	1.79 (0.99, 3.23)	1 (reference)	1.50 (0.88, 2.58)	1.76 (0.77, 4.04)	2.95 (1.63, 5.34)	0.07	
Women	13,233	116	1.37 (0.60, 3.11)	1.20 (0.60, 2.42)	1 (reference)	1.16 (0.60, 2.25)	1.06 (0.59, 1.91)	2.21 (1.06, 4.59)	0.24	0.66
Men	10,979	80	2.78 (0.77, 10.08)	3.27 (1.02, 10.48)	1 (reference)	2.61 (0.88, 7.72)	1.46 (0.85, 2.49)	5.21 (1.80, 15.10)	0.16	
Influenza and pneumonia mortality										
Total	24,212	88	1.11 (0.46, 2.67)	0.49 (0.18, 1.31)	1 (reference)	1.35 (0.64, 2.84)	0.86 (0.34, 2.17)	1.30 (0.58, 2.90)	0.23	
Women	13,233	49	1.95 (0.52, 5.20)	1.05 (0.30, 3.67)	1 (reference)	2.08 (0.78, 5.58)	1.80 (0.52, 6.22)	1.75 (0.66, 4.63)	0.44	0.46
Men	10,979	39	0.86 (0.22, 3.31)	0.22 (0.05, 0.97)	1 (reference)	0.98 (0.37, 2.63)	0.28 (0.06, 1.46)	0.88 (0.25, 3.09)	0.51	
Kidney disease mortality										
Total	24,212	135	2.20 (1.00, 4.84)	1.57 (0.59, 4.19)	1 (reference)	1.17 (0.59, 2.33)	3.15 (1.26, 7.85)	5.18 (2.18, 12.28)	0.05	
Women	13,233	69	2.26 (0.89, 5.75)	1.77 (0.54, 5.80)	1 (reference)	0.94 (0.38, 2.29)	3.74 (1.33, 10.49)	3.12 (0.99, 9.79)	0.63	0.37
Men	10,979	66	2.40 (0.73, 7.83)	1.20 (0.44, 3.28)	1 (reference)	1.44 (0.62, 3.37)	2.25 (0.53, 9.50)	8.16 (2.93, 22.87)	0.009	
Alzheimer’s disease mortality										
Total	24,212	74	0.90 (0.34, 2.42)	0.79 (0.34, 1.86)	1 (reference)	0.87 (0.40, 1.91)	1.34 (0.47, 3.85)	2.64 (1.14, 6.08)	0.013	
Women	13,233	49	0.53 (0.13, 1.64)	1.59 (0.64, 3.93)	1 (reference)	1.04 (0.37, 2.89)	2.24 (0.75, 6.64)	2.97 (1.15, 7.68)	0.028	0.30
Men	10,979	25	0.86 (0.26, 2.80)	0.15 (0.01, 1.96)	1 (reference)	0.59 (0.16, 2.22)	0.45 (0.05, 3.99)	1.80 (0.45, 7.25)	0.10	
CLRD mortality										
Total	24,212	202	1.21 (0.61, 2.41)	1.42 (0.82, 2.46)	1 (reference)	1.83 (1.08, 3.08)	1.41 (0.69, 2.91)	2.55 (1.42, 4.60)	0.014	
Women	13,233	109	0.63 (0.24, 1.46)	1.18 (0.58, 2.38)	1 (reference)	1.77 (0.88, 3.55)	1.10 (0.39, 3.14)	2.55 (1.42, 4.60)	0.002	0.50
Men	10,979	93	2.20 (0.84, 5.75)	1.73 (0.72, 4.17)	1 (reference)	1.94 (0.89, 4.24)	1.77 (0.65, 4.84)	2.62 (0.99, 6.90)	0.52	

^a^*p* value for interaction term is between sex and sleep duration

### Sleep duration and cause-specific mortality

People with type 2 diabetes who slept for ≤5 h/day, 8 h/day and ≥10 h/day had 41%, 26% and 59% greater risk of cancer mortality, respectively, compared with those who slept for 7 h/day (Table 3 and Fig. 4b). The association between sleep duration and CVD mortality risk in individuals with type 2 diabetes was only statistically significant for people who slept for ≥10 h/day (HR 1.74 [1.33, 2.28]) (Table 3 and Fig. 4c). For heart disease mortality specifically, the excess risk was only statistically significant for ≥10 h/day sleep duration (HR 1.51 [1.11, 2.07]). Of all the CVD types, the most prominent sleep–mortality association was observed for stroke mortality (sleep duration ≥10 h/day, HR 2.95 [1.63, 5.34]). For other causes of death, we observed a statistically significant association between sleep duration and mortality risk from kidney disease, Alzheimer’s disease and CLRD (Table 3). Although the subgroup analysis by sex showed higher HRs among men, the test for interaction term was not statistically significant due to wider CIs (Table 3).

### Subgroup analyses

#### Age of diabetes onset

Comparing people with type 2 diabetes diagnosed before the age of 45 years vs those diagnosed after 45 years, the shortest and longest sleep durations were associated with greatest risk of all-cause mortality relative to those sleeping 7 h/day among people (Table 4). For CVD-specific mortality, significant increased risk was only observed in those diagnosed with type 2 diabetes at the age of >45 years and who slept for ≥10 h/day (HR 1.73 [1.28, 2.33]) (Table 4).

**Table 4:** Subgroup analysis of the association of sleep duration with all-cause and CVD mortality among individuals with type 2 diabetes

Cause of mortality	Mortality risk according to sleep duration, HR (95% CI)						*p* trend	*p* interaction^a^
	≤5 h/day	6 h/day	7 h/day	8 h/day	9 h/day	≥10 h/day		
All-cause mortality								
Age at diagnosis, years								
≤45	1.37 (1.05, 1.79)	1.35 (1.06, 1.73)	1 (reference)	1.21 (0.95, 1.53)	1.78 (1.33, 2.37)	2.08 (1.61, 2.71)	0.007	<0.001
>45	1.19 (1.02, 1.38)	1.05 (0.92, 1.21)	1 (reference)	1.15 (1.03, 1.29)	1.21 (1.02, 1.43)	1.73 (1.50, 2.00)	<0.001	
Diabetes duration, years								
≤5	1.08 (0.85, 1.38)	1.17 (0.92, 1.48)	1 (reference)	1.08 (0.89, 1.31)	0.94 (0.69, 1.35)	1.68 (1.30, 2.18)	0.08	<0.001
6–10	1.28 (0.95, 1.72)	1.11 (0.85, 1.44)	1 (reference)	1.17 (0.93, 1.47)	1.47 (1.04, 2.10)	1.54 (1.14, 2.08)	0.05	
11–20	1.23 (0.95, 1.60)	1.01 (0.80, 1.27)	1 (reference)	1.19 (0.94, 1.43)	1.19 (0.89, 1.59)	2.18 (1.73, 2.74)	<0.001	
>20	1.41 (1.08, 1.84)	1.25 (0.98, 1.58)	1 (reference)	1.24 (1.00, 1.55)	1.72 (1.30, 2.27)	1.88 (1.49, 2.37)	0.001	
CVD mortality								
Age at diagnosis, years								
≤45	1.72 (0.97, 3.05)	1.29 (0.74, 2.25)	1 (reference)	1.31 (0.79, 2.15)	1.73 (0.89, 3.37)	1.58 (0.87, 2.86)	0.96	0.222
>45	1.00 (0.70, 1.41)	1.07 (0.81, 1.43)	1 (reference)	1.10 (0.86, 1.41)	1.23 (0.82, 1.86)	1.73 (1.28, 2.33)	0.002	
Diabetes duration, years								
≤5	0.75 (0.40, 1.40)	1.44 (0.89, 2.35)	1 (reference)	1.25 (0.79, 1.98)	1.61 (0.78, 3.34)	1.23 (0.65, 2.32)	0.26	0.054
6–10	0.58 (0.29, 1.15)	0.61 (0.35, 1.08)	1 (reference)	0.74 (0.44, 1.25)	1.05 (0.45, 2.47)	0.91 (0.48, 1.71)	0.12	
11–20	1.76 (0.98, 3.16)	1.36 (0.84, 2.21)	1 (reference)	1.34 (0.84, 2.13)	1.17 (0.59, 2.32)	2.98 (1.80, 4.95)	0.049	
>20	1.97 (1.08, 3.56)	1.27 (0.75, 2.12)	1 (reference)	1.40 (0.89, 2.19)	1.54 (0.85, 2.80)	1.98 (1.24, 3.17)	0.44	

^a^*p* value for interaction term is between age at diagnosis/diabetes duration and sleep duration

#### Duration of diabetes

Shorter sleep duration (≤5 h/day) was associated with the highest all-cause mortality risk in those living with type 2 diabetes for >20 years (HR 1.41 [1.08, 1.84]) (Table 4). For individuals who slept for ≥10 h/day, those living with type 2 diabetes for 11–20 years had an HR of 2.18 (95% CI 1.73, 2.74) and those living with type 2 diabetes for >20 years had an HR of 1.88 (95% CI 1.49, 2.37). Similar results were observed for CVD mortality when comparing against individuals who slept for 7 h/day. A more than 97% increased CVD mortality risk was detected in individuals living with type 2 diabetes for >20 years (sleep duration ≤5 h/day, HR 1.97 [1.08, 3.56]) and risk was increased in individuals who slept for ≥10 h/day (diabetes duration 11–20 years, HR 2.98 [1.80, 4.95]; diabetes duration >20 years, HR 1.98 [1.24, 3.17]).

#### Medication status

In individuals with type 2 diabetes treated with both oral glucose-lowering medication and insulin, higher HRs were observed for all-cause mortality in those who slept for shorter and longer durations (Table 5). People treated with both oral glucose-lowering medication and insulin also had greater HRs for CVD mortality risk according to sleep duration (≤5 h/day, HR 2.69 [1.35, 5.37]; ≥10 h/day, HR 2.80 [1.43, 5.47]) (Table 5).

**Table 5:** Subgroup analysis of the association of sleep duration with all-cause and CVD mortality by treatment

Cause of mortality	Mortality risk according to sleep duration, HR (95% CI)						*p* trend	*p* interaction^a^
	≤5 h/day	6 h/day	7 h/day	8 h/day	9 h/day	≥10 h/day		
All-cause mortality								
Oral glucose-lowering medication only	1.22 (1.03, 1.44)	1.08 (0.92, 1.27)	1 (reference)	1.14 (1.00, 1.29)	1.26 (1.03, 1.55)	1.70 (1.44, 2.02)	0.09	<0.001
Insulin only	1.03 (0.71, 1.49)	1.01 (0.75, 1.35)	1 (reference)	1.01 (0.77, 1.32)	1.28 (0.89, 1.85)	1.54 (1.15, 2.05)	0.053	
Both oral glucose-lowering medication and insulin	1.55 (1.09, 2.20)	1.20 (0.87, 1.66)	1 (reference)	1.28 (0.96, 1.71)	1.56 (1.07, 2.26)	1.80 (1.32, 2.45)	<0.001	
No medication	1.08 (0.72, 1.63)	1.34 (0.93, 1.92)	1 (reference)	1.26 (0.94, 1.70)	1.22 (0.72, 2.08)	2.24 (1.49, 3.36)	0.001	
CVD mortality								
Oral glucose-lowering medication only	0.97 (0.67, 1.41)	0.96 (0.69, 1.33)	1 (reference)	0.93 (0.70, 1.23)	1.10 (0.67, 1.80)	1.38 (0.96, 1.98)	0.26	0.001
Insulin only	1.04 (0.44, 2.43)	1.67 (0.88, 3.18)	1 (reference)	1.08 (0.09, 1.96)	1.68 (0.79, 3.58)	1.99 (1.01, 3.91)	0.12	
Both oral glucose-lowering medication and insulin	2.69 (1.35, 5.37)	1.30 (0.67, 2.52)	1 (reference)	1.87 (0.97, 3.63)	2.26 (0.99, 5.14)	2.80 (1.43, 5.47)	0.049	
No medication	0.93 (0.30, 2.88)	1.78 (0.81, 3.91)	1 (reference)	1.99 (0.99, 3.99)	2.06 (0.57, 7.39)	2.30 (0.92, 5.74)	0.44	

^a^*p* value for interaction term is between treatment and sleep duration

### Sensitivity analysis

Sensitivity analysis was performed to test the robustness of the main effects models. After eliminating participants with a history of CVD and/or cancer, results remained largely unchanged for all associations (ESM Table 3).

## Discussion

In this population-based prospective cohort study, we observed that the absolute mortality rate was higher in adults with diabetes and extremes of sleep duration. There was a non-significant interaction between sleep duration and the presence of diabetes (*p* for interaction = 0.08). Extremes of sleep duration were associated with significant increases in all-cause and disease-specific mortality risk among people with type 2 diabetes. The risk was greatest for those who reported the longest mean sleep duration. The associations between sleep duration and mortality from all causes, cancer, CVD, heart disease, stroke, kidney disease and CLRD appeared to be J-shaped while the association between sleep duration and Alzheimer’s disease mortality risk appeared to be most pronounced at ≥10 h/day. Subgroup analysis showed that these associations were most pronounced for people who were diagnosed with diabetes at a younger age and for those who reported living with diabetes for a longer duration and used both oral glucose-lowering medication and insulin. Our findings suggest that excessive or insufficient amounts of sleep may be risk factors for all-cause and CVD mortality in people with type 2 diabetes.

Although the association between sleep duration and all-cause mortality has been investigated in several studies among the general population [25–27], few studies have explored this association in individuals with established diabetes. This study provides evidence that sleep duration is associated with higher mortality risk among people with type 2 diabetes and that the absolute mortality rate is higher in diabetic individuals with extreme sleep duration. Previous studies have demonstrated the significant gap in mortality risk between adults with and without diabetes [9, 28]. The magnitude of effects of risk factors such as socioeconomic and lifestyle factors on survival outcomes may differ between the diabetic and non-diabetic population. A recent study showed that the impact of higher leisure-time physical activity was even stronger in adults with CVD than in those without [11]. The effect of relative educational disparities on mortality risk was demonstrated to differ in adults with and without diabetes [10], but no difference in the obesity–mortality association was found between those with and without diabetes [29]. In our study, the *p* for interaction was 0.08, indicating that there may be a difference in the association between sleep duration and mortality when comparing diabetic and non-diabetic individuals, but this did not reach significance.

Laboratory studies conducted in healthy adults have revealed that insufficient sleep is associated with 40% slower glucose clearance rate and higher sympathetic nervous system activity compared with a sleep recovery condition (mean sympathovagal balance 0.77 vs 0.66) [30]. Sympathetic activation may aggravate the status of insulin resistance, obesity and hypertension [31–33]. Therefore, sleep deprivation in people with type 2 diabetes is likely to exacerbate complications and affect the control and management of blood glucose, which drive excess mortality risk. It is acknowledged, however, that sleep is a complex phenomenon and extreme sleep duration may reflect poorer health status and reduced functioning (e.g. our finding that people with type 2 diabetes who sleep longer have greater mortality risk; such people may experience more severe diabetes-related complications that require more rest or long-term bed rest). Another possible explanation of these findings is that longer sleep duration has been associated with chronic inflammatory responses, which increase mortality risk. Indeed, IL-6 and C-reactive protein, indicators of infection, are elevated in individuals who report long sleep duration [34]. Chronic inflammation may accelerate the progressive condition of diabetes and its complications [35]. Recently, shorter sleep duration was shown to predict arterial stiffness in young to middle-aged adults [36].

Our findings further suggest that extremes of sleep duration (shorter or longer than 7 h/day) increase all-cause and CVD mortality in people with type 2 diabetes, particularly those with diabetes of a longer duration and who are diagnosed at an earlier age. Findings from other studies corroborate this. One recent study conducted among 3724 individuals with type 2 diabetes indicated that disease duration ≥10 years (vs <5 years) was associated with 1.82 and 1.48 times higher risk of all-cause mortality and heart failure, respectively [37]. Individuals with longer diabetes duration may be susceptible to burden or fatigue associated with chronic disease management. Similarly, those diagnosed at an early age may have a poorer overall health profile for other chronic conditions as well as that pertaining to their diabetes. This may include higher BMI, disease-related complications, medication adherence and poorer blood glucose control when compared with individuals having a shorter diabetes duration and older age at onset of diabetes [38]. Diabetes-related distress often experienced by younger people with type 2 diabetes may exacerbate problems maintaining diabetes control and other areas of self-management including sleep hygiene. However, evidence for the effectiveness of sleep interventions for improving clinical outcomes in people with type 2 diabetes is complex. This is exemplified by interventions to relieve sleep apnoea, an established risk factor for CVD events and mortality. On one hand, continuous positive airway pressure (CPAP) over 6 months was shown to improve glycaemic control and insulin resistance in people with type 2 diabetes and obstructive sleep apnoea [39] but on the other, one earlier trial of CPAP over 3 months found no such benefits [40].

In our study, subgroup analysis based on medication status showed that diabetic individuals with shorter sleep duration have the highest risk for mortality when they are treated with both insulin and oral glucose-lowering medication. A previous study showed that insulin-treated individuals with type 2 diabetes had higher Pittsburgh Sleep Quality Index and poor sleep quality [38]. Another study found that metformin therapy was associated with longer sleep duration and better sleep efficiency than that in patients not treated with metformin [39]. Patients with type 2 diabetes using both insulin treatment and oral glucose-lowering medication may have poorer glucose metabolism and glycaemic control and poorer sleep quality than those not using these medications. In addition, for individuals who have type 2 diabetes and short sleep duration, those using both insulin treatment and oral glucose-lowering medication have poorer sleep quality, such as obstructive sleep apnoea [17]. This poor sleep quality may partly explain the results that sleep deprivation was associated with the highest mortality risk among individuals with type 2 diabetes using both insulin and oral glucose-lowering medication [41].

Our study provides quantitative estimates regarding the association between sleep duration and all-cause and cause-specific mortality in people with type 2 diabetes. For people with type 2 diabetes, as per the general population, 6–8 h of sleep is recommended for reducing mortality risk [13, 26]. Sleep interventions as an adjunct to standard diabetes treatment may warrant further attention. Monitoring of sleep duration may serve as a useful tool for identifying high-risk people with type 2 diabetes in clinical practice for possible intervention, especially for those being treated with both oral glucose-lowering drugs and insulin.

Our study has several strengths, including the relatively large sample size. In addition, we conducted subgroup and sensitivity analyses to ensure the robustness of the study findings. Several limitations should also be noted. First, sleep duration and diabetes history were determined from a self-reported questionnaire without using any objective measurements. However, any measurement errors in the assessment of sleep duration would most likely be non-differential and lead to underestimation of the observed associations because of the prospective design of the study [42]. Second, the present study did not include non-fatal events, which limited our ability to estimate risk of incident disease. Third, Stata have not provided the procedure of complex survey design data to account for competing risk, thus HR estimates may be overestimated due to estimation bias caused by ignoring competing risks. Fourth, due to the nature of observational studies, the current study cannot infer causality of the sleep–mortality association among people with type 2 diabetes. Finally, we lacked data on sleep quality and other factors that may be responsible for deterioration of sleep quality (e.g. number/age of young children, type of employment, medications, anxiety/depression, sleep apnoea and insomnia). This precludes in-depth analysis in relation to sleep problems and mortality among people with type 2 diabetes. Further studies in this area should concentrate on addressing such issues.

In summary, there is some preliminary evidence that the associations between sleep duration and mortality differ between diabetic and non-diabetic individuals. Diabetes patients sleeping for less than or in excess of 7 h conferred an increased risk of all-cause and cause-specific mortality. The association was more prominent in those with younger age at disease onset. These individuals may require greater medical attention that targets sleep and lifestyle to reduce the risks of adverse health outcomes.

## Electronic supplementary material

ESM Tables (PDF 276 kb)

## Data Availability

The NHIS data are available from www.cdc.gov/nchs/nhis/index.htm

## Abbreviations

CLRD: Chronic lower respiratory disease
CPAP: Continuous positive airway pressure
NCHS: National Center for Health Statistics
NDI: National Death Index
NHIS: National Health Interview Survey
