# Supplementary material for: Association between sleep duration and mortality risk among adults with type 2 diabetes: a prospective cohort study
Source: Diabetologia. 2020 Jul 16;63(11):2292–304. doi: 10.1007/s00125-020-05214-4 (PMC7527363; doi:10.1007/s00125-020-05214-4)
Supplement: Supplementary file 1 — (PDF 276 kb) [file 125_2020_5214_MOESM1_ESM.pdf]

Electronic supplementary material

ESM Table 1. ICD codes for specific causes of death

	ICD10
All - CVD	I00-I09, I11, I13, I20-I51, I60-I69
Heart disease	I00-I09, I11, I13, I20-I51
Stroke	I60-I69
All cancer	C00-C97
Chronic lower respiratory diseases	J40-J47
Alzheimer's disease	G30
Diabetes mellitus	E10-E14
Influenza and pneumonia	J09-J18
Nephritis, nephrotic syndrome and nephrosis	N00-N07, N17-N19, N25-N27

Reference. The 113 underlying cause of death recode were used to regroup the specific mortality according to the NCHS.

ESM Table 2. Baseline characteristics among non-diabetes population according to sleep duration.

Baseline Characteristics	Sleep duration (h/d)					
	≤ 5	6	7	8	9	≥10
Sample size	20,676 (7.8)	52,316 (20.8)	75,182 (31.0)	79,669 (32.2)	11,313 (4.6)	9,661 (3.8)
Mean age, (years)	46.49	45.63	45.47	46.68	48.86	52.14
Sex (%)						
Male	8,760 (46.9)	23,563 (49.3)	34,671 (49.9)	34,595 (47.0)	4,505 (43.3)	4,010 (45.5)
Female	11,916 (53.1)	28,753 (50.7)	40,511 (50.2)	45,074 (53.0)	6,808 (56.7)	5,651 (54.5)
Race (%)						
Hispanic	3,278 (12.2)	8,381 (12.5)	12,526 (12.8)	15,956 (15.7)	2,052 (14.3)	1,668 (13.9)
Non-Hispanic White	11,887 (66.0)	31,418 (68.1)	48,787 (72.6)	48,295 (68.6)	7,238 (71.4)	5,585 (65.8)
Non-Hispanic Black	4,342 (16.8)	8,989 (13.4)	8,781 (8.9)	10,808 (10.5)	1,497 (10.0)	1,996 (16.5)
Non-Hispanic Other	1,169 (5.0)	3,528 (6.0)	5,088 (5.7)	4,610 (5.2)	562 (4.4)	412 (3.8)
Education level (%)						
Less than high school degree	4,059 (17.6)	7,524 (12.7)	9,409 (10.9)	14,437 (15.8)	2,363 (18.2)	2,854 (27.9)
High school degree	6,014 (30.4)	13,878 (27.1)	17,926 (24.1)	21,876 (28.1)	3,362 (31.4)	3,187 (34.3)
More than high school degree	10,510 (51.5)	30,727 (59.8)	47,504 (64.4)	42,878 (55.4)	5,540 (50.0)	3,529 (36.8)
Income (%)						
Low	4,780 (18.1)	8,327 (12.0)	9,647 (9.5)	13,504 (12.9)	2,236 (15.4)	2,589 (21.7)
Middle	10,805 (52.6)	26,392 (49.2)	35,816 (45.6)	40,262 (49.0)	6,072 (52.4)	5,374 (56.7)
High	5,091 (29.4)	17,597 (38.8)	29,719 (44.9)	1,763 (38.1)	3,005 (32.1)	1,698 (21.6)
BMI (kg/m2) (%)						
Normal weight/Underweight, <25 kg/m2	6,773 (32.3)	18,612 (35.4)	29,923 (39.5)	32,057 (40.9)	4,722 (43.0)	3,917 (42.1)
Pre-obesity, 25-30 kg/m2	6,768 (33.0)	17,959 (34.6)	26,589 (35.5)	27,361 (34.3)	3,712 (32.1)	2,885 (29.6)
Obesity, >30 kg/m2	6,577 (32.0)	14,358 (27.5)	16,658 (22.3)	17,918 (21.9)	2,600 (22.5)	2,586 (25.9)
Physical activity (meeting,%)	7,870 (39.9)	23,200 (45.6)	36,057 (49.3)	24,296 (44.9)	4,530 (41.9)	2,605 (28.7)
Smoking status (%)						
Never	10,284 (49.0)	29,730 (56.1)	46,796 (62.0)	48,893 (61.4)	6,467 (57.6)	4,846 (50.0)
Former smoker	4,001 (19.5)	10,635 (20.6)	15,425 (20.7)	16,242 (20.4)	2,590 (22.2)	2,386 (23.7)
Current smoker	6,365 (31.4)	11,871 (23.2)	12,827 (17.1)	14,334 (17.9)	2,245 (20.2)	2,407 (26.1)
Hypertension (%)	7,003 (31.5)	14,443 (25.7)	17,598 (22.1)	20,343 (23.3)	3,482 (27.6)	3,604 (33.4)
CHD (%)	1,086 (4.7)	1,808 (3.1)	2,170 (2.7)	2,858 (3.3)	567 (4.6)	786 (7.6)
Stroke (%)	737 (3.2)	1,109 (1.8)	1,089 (1.2)	1,718 (1.8)	423 (3.4)	779 (7.5)
Cancer (%)	1,818 (8.4)	3,682 (6.7)	4,993 (6.4)	6,066 (7.2)	1,152 (9.6)	1,231 (12.1)

Values are n (percentage).
Abbreviation: BMI, body mass index, CHD, coronary heart disease

ESM Table 3. Sensitivity analysis excluding individuals with CHD, stroke and cancer for the association of sleep duration with all-cause and cause-specific mortality.

Outcomes	N. of participants	N. of Deaths	Sleep duration (h/d)					
			≤ 5	6	7	8	9	≥10
All-cause mortality	16,014	2,153	1.28 (1.06,1.55)	1.22 (1.03,1.45)	1(ref.)	1.04 (0.89,1.21)	1.38 (1.11,1.70)	1.73 (1.41,2.13)
Cancer mortality	16,014	456	1.40 (0.92,2.12)	1.11 (0.76,1.60)	1(ref.)	1.12 (0.82,1.53)	1.26 (0.80,1.99)	1.23 (0.77,1.97)
CVD mortality	16,014	387	1.25 (0.77,2.02)	1.45 (0.99,2.13)	1(ref.)	1.04 (0.70,1.54)	1.54 (0.90,2.64)	1.87 (1.20,2.92)
Heart disease mortality	16,014	299	0.91 (0.51,1.63)	1.30 (0.82,2.07)	1(ref.)	0.94 (0.60,1.47)	1.58 (0.87,2,89)	1.69 (1.01,2.82)
Stroke mortality	16,014	88	2.95 (1.15,7.61)	2.21 (0.95,5.14)	1(ref.)	1.51 (0.66,3.35)	1.42 (0.41,4.96)	2.70 (1.12,6.49)
Influenza and Pneumonia mortality	16,014	44	1.54 (0.46,5.12)	0.16 (0.02,1.30)	1(ref.)	1.24 (0.44,3.50)	0.82 (0.22,3.12)	0.92 (0.26,3.34)
Kidney disease mortality	16,014	68	1.94 (0.78,4.80)	2.07 (0.81,5.26)	1(ref.)	0.67 (0.27,1.64)	1.94 (0.55,6.85)	4.70 (0.95,23.22)
Alzheimer’s disease mortality	16,014	35	2.58 (0.57,11.65)	1.11 (0.21,5.89)	1(ref.)	1.11 (0.23,5.45)	1.94 (0.36,10.61)	4.70 (0.95,23.22)
CLRD mortality	16,014	94	1.05 (0.35,3.13)	1.69 (0.73,3.95)	1(ref.)	2.23 (1.03,4.28)	1.90 (0.66,5.47)	3.26 (1.39,7.64)

Values are HR (95% CI);
Abbreviation: CHD, coronary heart disease; CI, confidence interval; CLRD, chronic lower respiratory diseases; CVD, cardiovascular disease; HR, hazard ratio, N., number.
